# The Clinical and Polynucleotide Repeat Expansion Analysis of *ATXN2, NOP56, AR* and *C9orf72* in Patients With ALS From Mainland China

**DOI:** 10.3389/fneur.2022.811202

**Published:** 2022-05-06

**Authors:** Xiaorong Hou, Wanzhen Li, Pan Liu, Zhen Liu, Yanchun Yuan, Jie Ni, Lu Shen, Beisha Tang, Junling Wang

**Affiliations:** ^1^Department of Neurology, Xiangya Hospital, Central South University, Changsha, China; ^2^National Clinical Research Center for Geriatric Diseases, Xiangya Hospital, Central South University, Changsha, China; ^3^Key Laboratory of Hunan Province in Neurodegenerative Disorders, Central South University, Changsha, China; ^4^Laboratory of Medical Genetics, Central South University, Changsha, China; ^5^Engineering Research Center of Hunan Province in Cognitive Impairment Disorders, Central South University, Changsha, China; ^6^Hunan International Scientific and Technological Cooperation Base of Neurodegenerative and Neurogenetic Diseases, Changsha, China

**Keywords:** amyotrophic lateral sclerosis, nucleotide repeat expansion, neurodegenerative disease, *ATXN2*, *C9orf72*

## Abstract

**Background:**

Repeat expansions, including those in *C9orf72* and *ATXN2*, have been implicated in amyotrophic lateral sclerosis (ALS). However, there have been few studies on the association of *AR* and *NOP56* repeat expansion with ALS, especially in China. Accordingly, we aimed to evaluate the frequency of *C9orf72* and *ATXN2* repeat mutations and investigate whether *NOP56* and *AR* repeat expansion are risk factors for ALS.

**Methods:**

In this study, 736 ALS patients and several hundred healthy controls were recruited. Polymerase chain reaction (PCR) and repeat-primed PCR (RP-PCR) were performed to determine the repeat lengths in *C9orf72, ATXN2, AR*, and *NOP56*.

**Results:**

GGGGCC repeats in *C9orf72* were observed in six ALS patients (0.8%, 6/736) but not in any of the controls (0/365). The patients with pathogenic GGGGCC repeats showed shorter median survival times than those with a normal genotype (*p* = 0.006). Regarding *ATXN2* CAG repeats, we identified that intermediate repeat lengths (29–34 copies) were associated with ALS (*p* = 0.033), and there was no difference in clinical characteristics between the groups with and without intermediate repeats (*p* > 0.05). Meanwhile, we observed that there was no association between the repeat size in *AR* and *NOP56* and ALS (*p* > 0.05).

**Conclusions:**

Our results demonstrated that pathogenetic repeats in *C9orf72* are rare in China, while intermediate CAG repeats in *ATXN2* are more frequent but have no effect on disease phenotypes; the repeat size in *AR* and *NOP56* may not be a risk factor for ALS.

## Introduction

Amyotrophic lateral sclerosis (ALS) is a progressive neurodegenerative disease that affects upper and lower motor neurons and results in muscular weakness and atrophy (1). To date, mutations in more than 50 genes have been associated with the pathogenesis of ALS (2). Notably, nucleotide repeat expansion mutations in part of these genes play a pivotal part in the pathogenetic process. For example, the pathogenic GGGGCC hexanucleotide repeat expansion (HRE) in the chromosome 9 open reading frame 72 (*C9orf72*) gene was identified as a common causative factor for ALS in Caucasian populations, accounting for 23.5–47% of familial ALS (3, 4) and 4.1–21.0% of sporadic ALS cases (5). However, this mutation was very rare in mainland China and other Asian countries (6–8), with a very low frequency of only 0%-0.3%. As early as 2010, intermediate-length CAG repeats (27–33 copies) in the *ATXN2* gene were reported as a genetic risk factor for ALS (9). Subsequently, many studies have validated this result in many other ethnicities (10–14).

Spinal and bulbar muscular atrophy (SBMA), a neuromuscular disease that shares several clinical features with ALS, is caused by CAG trinucleotide repeat expansions (>38 copies) in the androgen receptor (*AR*) gene (15). A functional study showed that AR was downregulated in the spinal cord in male SOD1^G93A^ mice, suggesting that AR may play an important role in the pathogenesis of ALS (16). Another nucleotide repeats expansion disease, spinocerebellar ataxia type 36 (SCA36), caused by GGCCTG hexanucleotide repeat expansion in the *NOP56* gene, also presents an ALS phenotype (17, 18). Subsequent studies revealed motor neuron involvement during the course of SCA36, including reduced expression of NOP56, TDP-43, and FUS in the large motor neurons of an ALS mouse model, which occurred before the apparent onset of the disease (19). Nonetheless, few studies have been conducted to date on the association between ALS and the size of nucleotide repeats within *AR* or *NOP56*.

Therefore, in this study, we aimed to determine (i) the frequency of *C9orf72* and *ATXN2* mutations; (ii) whether *NOP56* and *AR* are risk genes for ALS; and (iii) the potential associations between phenotypes and the size of nucleotide repeats within the *C9orf72, ATXN2, NOP56*, and *AR* genes.

## Methods

### Population

ALS patients were enrolled from the Department of Neurology, Xiangya Hospital, Central South University (CSU), in either an outpatient or an inpatient setting from April 30, 2013, to November 30, 2020. All patients were diagnosed by at least two experienced senior neurologists and diagnosed with clinically definite, probable, or probable laboratory-supported ALS according to the revised El Escorial criteria (20). Among the 736 patients, pathogenic mutations in known ALS-causative genes were excluded by whole-exome sequencing. This study also enrolled different numbers of healthy controls (365 controls in the *C9orf72* study, 201 controls in the *ATXN2* study, 225 controls in the *AR* study, and 229 controls in the *NOP56* study) from the Health Management Center of Xiangya Hospital, Central South University. Written informed consent was obtained from all participants, as approved by the Ethics Committee and the Expert Committee of Xiangya Hospital, Central South University.

### Genetic Analysis

Genomic DNA from all participants was extracted from peripheral blood using a standard extraction method.

To detect the size of repeat expansions in the *C9orf72* and *NOP56* genes, we first applied the polymerase chain reaction (PCR) method as described previously (8, 18). The target sequences covering the hexanucleotide repeats of the *C9orf72* and *NOP56* genes were amplified with different pairs of fluorescently labeled primers. The fluorescent PCR product was analyzed using an ABI-Prism 3730 Genetic Analyzer, and the data were examined using GeneMapper software (Applied Biosystems, Vernon Hills, Illinois, USA). Next, if the result showed a homozygous peak, we reanalyzed it using repeat-primed PCR (8, 18). Expansions of the hexanucleotide repeat showed a typical sawtooth pattern.

Genotyping of *ATXN2* and *AR* was performed by PCR amplification of CAG tracts in combination with capillary electrophoresis; the analysis was performed using GeneMarker software as described previously (21, 22).

### Statistical Analysis

For CAG repeats in *ATXN2*, we used the Fisher's exact test to assess the association between the intermediate CAG repeats and ALS. To identify the relationship between the repeat size in *AR* and *NOP56* and ALS, we used one-sided Mann-Whitney and outlier analysis, the same statistical methods as the tool ExpansionHunter DeNovo (23).

In ALS patients, the chi-square test, Spearman's or Fisher's exact test (for categorical variables) and the Mann-Whitney U test, Pearson's tests or Student's *t*-test (for continuous variables) were used to measure the association between polynucleotide repeat expansion and different clinical characteristics, such as age at onset (AAO), sex, site of onset, family history, Medical Research Council (MRC) score, Amyotrophic Lateral Sclerosis Functional Rating Scale-Revised (ALSFRS-R) score and disease progression rate (DPR; calculated as DPR = [48-ALSFRS-R score at time of diagnosis)/disease duration (months)] (24).

Kaplan-Meier univariate analysis was used to determine the effect of polynucleotide repeat expansion on survival time.

Statistical analyses were performed using IBM Statistical Package for the Social Sciences (SPSS) version 22. Differences with *P* < 0.05 were considered statistically significant.

## Results

### Clinical Data

A total of 736 ALS patients (679 with sporadic ALS and 57 with familial ALS) were recruited, including 486 males and 250 females. In our ALS cohort, the mean AAO was 52.44 ± 11.81 years; the percentage of patients exhibiting spinal onset (77%, 567/736) was higher than the percentage of patients with bulbar onset (22.9%, 169/736). Among 569 patients with ALSFRS-R data available, the mean score was 37.64 ± 7.95, and the mean DPR was 0.96 ± 0.97; among 663 patients with MRC scores available, the mean score was 107.97 ± 17.60.

### Genetic Results and Clinical Features of ALS Patients With *C9orf72* Mutations

**Figure 1:**
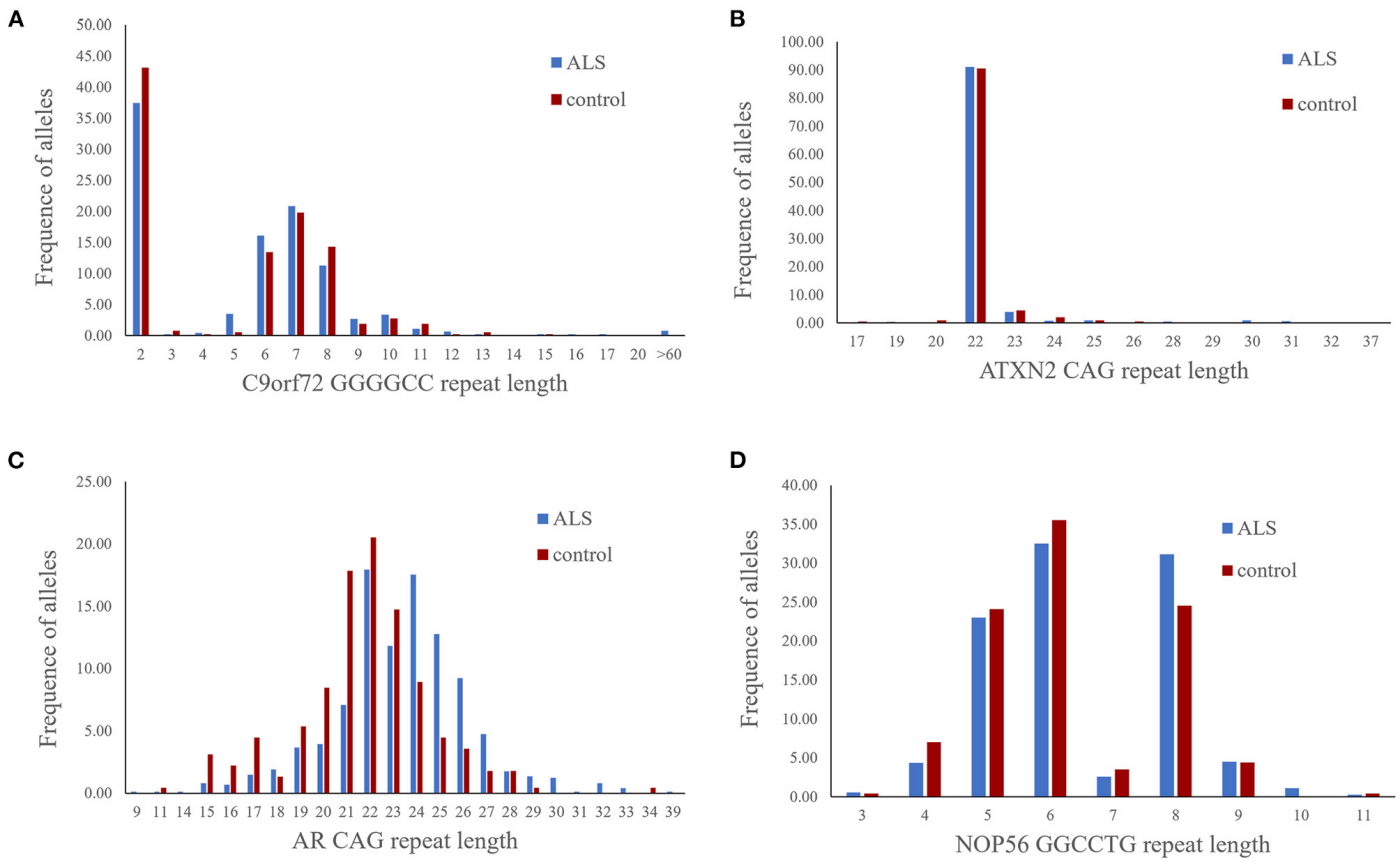
Comparison of repeat lengths in the long alleles of *C9orf72* **(A)**, *ATXN2* **(B)**, *AR* **(C)**, and *NOP56* **(D)** between ALS patients and controls.

The distribution of GGGGCC HRE length is shown in Figure 1A. Among 736 ALS patients, the GGGGCC HRE length ranged from two to more than 60 copies. The most frequent size of allele was two repeats, accounting for 37.3% of cases. Notably, we identified HREs over 30 repeats long in six ALS patients, accounting for 0.8%, but not in any of the controls (Table 1). Among the 365 healthy controls, the HRE length ranged from two to 15 copies; as in the patient group, the most common repeat length was two copies, accounting for 42.7% of cases. No intermediate-length HRE repeats (24–30) were identified in ALS patients or controls.

**Table 1:** Analysis of GGGGCC HREs in the *C9orf72* gene in ALS patients and controls.

***C9orf72***	**ALS patients**	**Healthy controls**	***p*-value**
Number	736	365	
Repeat range (most common size)	2–>60 (2)	2–15 (2)	
Mean ± SD	10.12 ± 5.27	5.2 ± 4.1	
Group			/^†^
<30, number	730	365	
≥30, number	6	0	

*SD, standard deviation.*

† *We did not do the Fisher's exact test as C9orf72 is a recognized disease-causing gene for ALS*.

**Figure 2:**
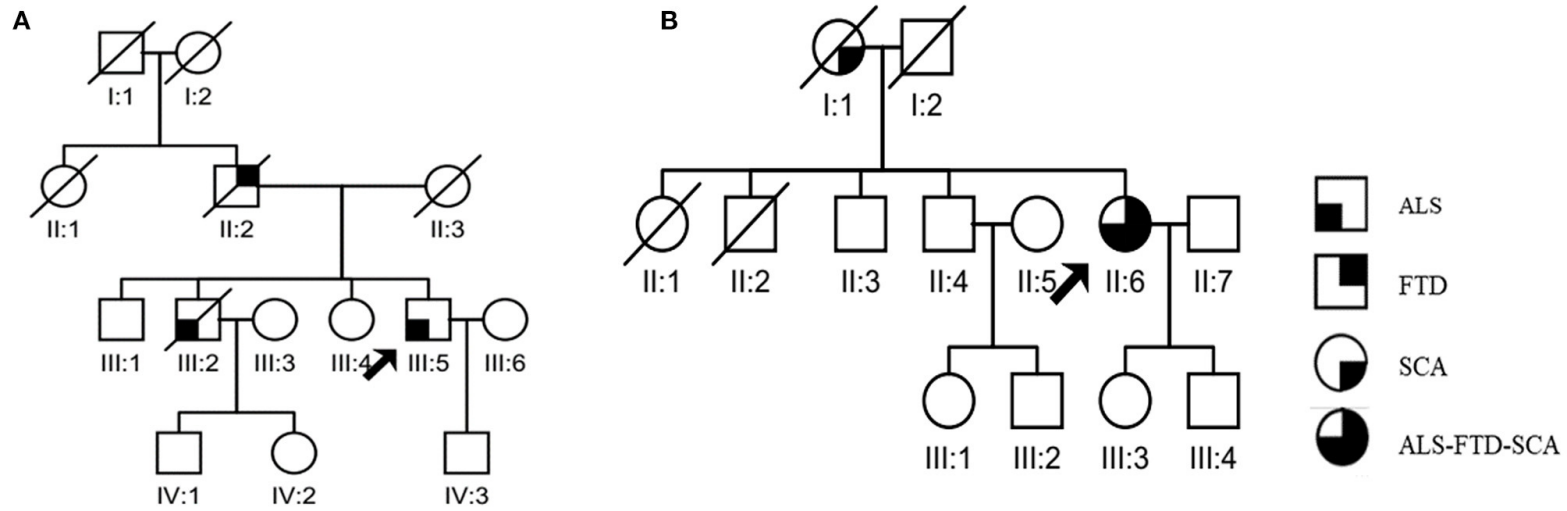
Pedigrees of ALS patients carrying an HREs mutation in *C9orf72* **(A)** and a CAG repeat of length 37 in *ATXN2* **(B)**.

The clinical features of the patients with HRE length ≥30 copies are shown in Table 2. When comparing the clinical characteristics of ALS patients with different ranges of HRE length, we found that patients with HRE length ≥30 copies had a shorter median survival time than those with <30 copies (*p* = 0.006). Although there was no significant difference in sex, AAO, ALSFRS-R, site of onset, family history, MRC or DPR (*p* > 0.05), we noted that ALS patients with HRE length ≥30 copies tended to have a younger mean AAO (46.25 ± 2.22 years) than those with <30 copies (52.51 ± 11.75 years). Among the six ALS patients with ≥30 copies, two patients had a family history. One of the families had been reported in our previous study (8). In another family (Figure 2A), the proband was a 44-year-old man who initially presented progressive muscle weakness in the bilateral upper limbs. Thereafter, he progressively manifested muscle weakness and atrophy in all four extremities, along with dysphagia and dysarthria. On admission 12 months after onset, neurological examination revealed fasciculations, muscle weakness and hyperreflexia in the affected limbs. Electromyography (EMG) showed abundant and diffuse ongoing denervation and chronic reinnervation changes. The patient's Mini-Mental State Examination (MMSE) score was 29 of 30, and his Montreal Cognitive Assessment (MoCA) score was 25 of 30. This patient was diagnosed with laboratory-supported probable ALS according to the El Escorial criteria. Follow-up revealed that he died of respiratory failure 18 months after the onset of the neurological symptoms. His father had been diagnosed with clinical frontotemporal dementia (FTD) and died at 52 years of age; his brother had been diagnosed with ALS at 36 and died of respiratory insufficiency at 39 years of age 36 months after disease onset, but further details were unavailable.

**Table 2:** Clinical details of patients carrying *C9orf72* HREs.

**Patient**	**A20863^†^**	**A36387**	**A36905**	**A000232**	**A002555**	**A002681**
HRE repeat length	>60	>60	>60	>60	>60	>60
Sex	Male	Female	Male	Female	Male	Male
AAO (years)	47	49	45	44	67	49
Disease duration (months)	10 ^¶^	NA	17^‡^	24	37	15^‡^
Site of onset	Spinal	Bulbar	Spinal	Spinal	Spinal	Spinal
Muscle weakness and atrophy	+	+	+	+	+	+
Muscle fasciculation	+	+	+	+	+	-
Dysarthria	+	+	+	-	+	+
Dysphagia	+	-	+	-	+	+
Memory impairment	-	-	-	+	+	-
Abnormal behavior	-	-	-	+	+	-
Sensory disturbance	-	-	-	-	-	-
Pyramidal signs	+	-	+	+	+	+
Family history	+	-	+	-	-	-
EMG						
NCV	-	NA	-	-	+^§^	NA
SP	+	NA	+	+	+	NA
MUP	↑	NA	↑	↑	↑	NA
Brain atrophy (MRI)	-	NA	+	+	+	NA
MRC	106/130	130/130	100/130	112/130	102/130	91/130
MMSE	28/30	NA	30/30	19/30	NA	NA
ECAS	NA	NA	NA	NA	60/136	NA
ALSFRS-R	NA	NA	39/48	39/48	44/48	14/48

*NA, data not available; AAO, age at onset; EMG, electromyography; NCV, nerve conduction velocity; SP, spontaneous potentials; MUP, motor unit potentials; MRI, magnetic resonance imaging; MRC, Medical Research Council; MMSE, Mini-Mental State Examination; ALSFRS-R, ALS Functional Rating Scale–Revised; +, affected; -, unaffected.*

† *The patient has been reported in a previous study.*

𠈁 *The patient was dead at the time of the study.*

§ *Right peroneal nerve motor conduction did not produce a positive waveform.*

¶ *Lost to follow-up*.

### Genetic Results and Clinical Features of ALS Patients With Intermediate CAG Repeats in *ATXN2*

As shown in Figure 1B, the CAG repeat length ranged from 17 to 37 in 736 ALS patients and from 17 to 26 in 201 controls. The most frequent allele size was 22 copies, accounting for 91.0 and 90.5% of ALS patients and controls, respectively. Herein, according to previous reports and a meta-analysis of *ATXN2* repeat lengths and ALS risk (31), we applied cutoff values and defined the following ranges: >34 copies as pathogenic expansion, <29 as normal repeats, and 29–34 repeats as intermediate repeats. We identified intermediate CAG repeats in 14 (1.90%, 14/736) ALS patients and none of the controls (*p* = 0.033) (Table 3, Supplementary Table 1).

**Table 3:** Analysis of intermediate-length CAG repeats in the *ATXN2* gene in ALS patients and controls.

***ATXN2***	**ALS patients**	**Healthy controls**	***p*-value**
Number	736	201	
Repeat range (most common size)	19–37 (22)	17–28 (22)	
Mean ± SD	25.93 ± 5.59	22.1 ± 0.8	
Group			0.033^‡^
<29, number	720	201	
29–34, number	14	0	

‡ *P-values were calculated via the Fisher's exact test*.

When comparing clinical characteristics between patients with intermediate repeat lengths and those with normal repeat lengths, we found that there was no significant difference in sex, AAO, ALSFRS-R, site of onset, MRC, familial history or DPR (*p* > 0.05). Using the Kaplan-Meier method, we did not find a significant difference in survival time between the two groups (*p* > 0.05).

**Figure 3:**
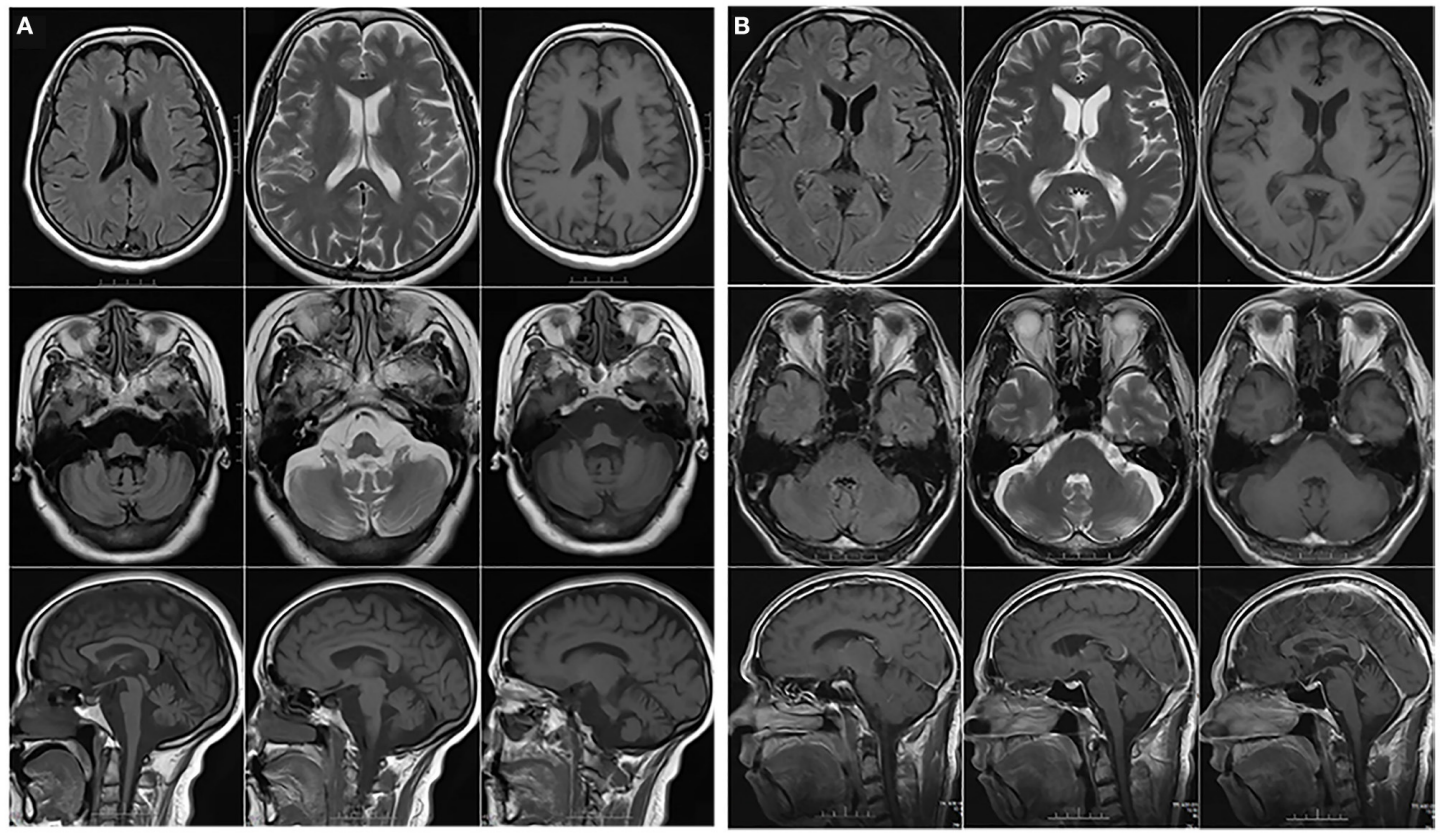
Brain magnetic resonance imaging from patient A0154 **(A)** and patient A002447 **(B)**.

We found two ALS patients carrying pathogenic CAG repeat expansions. Patient A0154, who carried 37 repeats, was a 52-year-old female with a family history of SCA (Figure 2B). She initially developed gait ataxia at 43 years old. Six years later, she gradually showed muscle weakness and atrophy of the bilateral lower limbs. At the age of 51 years, a neurological examination revealed obvious ataxia; the Romberg sign, finger-to-nose test, rapid alternating movements, and heel-to-shin test were positive. She also showed UMN signs such as a positive palmomental reflex and positive Babinski sign, as well as lower motor neuron (LMN) signs such as fasciculations, muscle atrophy and decreased pharyngeal reflex. EMG showed abundant and diffuse ongoing denervation and chronic reinnervation changes. Notably, the patient also clearly exhibited memory impairment. Her MMSE score was 18, and her Edinburgh Cognitive and Behavioral ALS Screen (ECAS) score was 42, suggesting cognitive impairment (executive dysfunction), memory impairment, and behavioral impairment (apathy, disinhibition, and loss of sympathy). Brain magnetic resonance imaging (MRI) revealed atrophy of the pons, cerebellum, and temporal lobe (Figure 3A). The patient was clinically diagnosed with SCA combined with probable ALS and behavioral-variant FTD (bvFTD). However, her mother presented symptoms of ataxia without any symptoms of ALS or FTD at 53 years old and died 20 years after onset.

Patient A002447, a 64-year-old male with a repeat length of 37, was from a family with no history of neurodegenerative diseases. He presented with dysarthria at the age of 62 years and then gradually developed weakness and atrophy in the right upper limb, which subsequently spread to the lower limbs in 6 months. Deep tendon reflexes were brisk, and the palmomental reflex was present. His MMSE (26/30), MoCA (16/30) and ECAS (50/136) revealed memory impairment and language dysfunction (difficulties in word finding and word comprehension). His brain MRI showed cortex atrophy, especially in the frontal and temporal lobes (Figure 3B). EMG showed abundant and diffuse ongoing denervation and chronic reinnervation changes. As mentioned above, he was clinically diagnosed with probable ALS together with semantic dementia.

### Genetic and Clinical Features of ALS Patients With Different CAG Repeats in the *AR* Gene

The sizes of CAG repeats in *AR* ranged from 9 to 39, with 22 repeats as the most common allele (17.9%) in 736 ALS patients; among the 225 healthy controls, the sizes ranged from 11 to 34, with 22 repeats as the most common allele (20.4%) (Figure 1C). In the male patients and controls, the repeat sizes of the long allele between the ALS and healthy control groups showed no significant difference (*n* = 610, Mann-Whitney U test, *p* = 0.315). Meanwhile, we found no association between the repeat sizes of the long allele and the risk for ALS in female patients and controls (*n* = 351, Mann-Whitney U test, *p* = 0.077). In addition, according to the outlier analysis, we applied 24 repeats and 28 repeats as the cutoff value in male and female patients, respectively. Statistically, CAG repeats in *AR* were not associated with ALS for each gender (Chi-square test, *p* > 0.05) (Table 4).

**Table 4:** Analysis of CAG repeats in the *AR* gene in ALS patients and controls.

***AR***	**ALS patients**	**Healthy controls**	***p*-value**
Number	736	225	
Repeat range (most common size)	9–39 (22)	11–34 (22)	
Mean ± SD	23.46 ± 7.75	21.8 ± 3.1	
Male <24, number	286	84	0.77^§^
≥ 24, number	200	40	
Female <28, number	26	5	0.690^§^
≥ 28, number	224	96	

§ *P-values were calculated via the chi-square test and Fisher's exact test*.

Next, regarding the association between CAG repeat size in *AR* and the ALS phenotypes, we found that repeat size did not affect the AAO, location of onset, family history, ALSFRS-R, MRC, DPR or survival time of ALS patients (*p* > 0.05).

### Genetic and Clinical Features of ALS Patients With Different GGCCTG Repeat Sizes in the *NOP56* Gene

The sizes of the GGCCTG repeats in *NOP56* ranged from three to 11 in 736 ALS patients and from three to nine in 229 controls (Figure 1D). Firstly, we used Mann-Whitney U test to test whether the repeat sizes of the long allele are associated with ALS. However, the repeat sizes of the long allele between the ALS and healthy controls showed no significant difference (*n* = 965, Mann-Whitney U test, *p* = 0.069). Meanwhile, we applied nine as the cutoff value according to the outlier analysis, and a repeat size above this value was not a risk factor for ALS (Chi-square test, *p* > 0.05) (Table 5). In addition, we found no significant associations in sex, AAO, family history, ALSFRS-R, site of onset, MRC or DPR between these two groups (*p* > 0.05). Moreover, we did not find any correlation between repeat length and survival time (*p* > 0.05).

**Table 5:** Analysis of GGCCTG repeats in the *NOP56* gene in ALS patients and controls.

***NOP56***	**ALS patients**	**Healthy controls**	***p*-value**
Number	736	229	
Repeat range (most common size)	9–39 (22)	11–34 (22)	
Mean ± SD	23.46 ± 7.75	21.8 ± 3.1	
Group			0.624^§^
<9, number	693	218	
≥9, number	43	11	

§ *P-values were calculated via the chi-square test*.

## Discussion

In this study, we systematically evaluated polynucleotide repeats in *C9orf72, ATXN2, AR*, and *NOP56* in a large Chinese ALS cohort and healthy controls. We found that six of 736 ALS patients (0.8%) carried a GGGGCC HRE in *C9orf72*. This frequency was similar to the rates observed in other Asian countries (0–4.7%) (25, 26) but was much lower than those in European populations (4.1–47%) (27). In addition, many studies have shown that patients carrying pathogenic HREs have phenotypic differences from those with normal HREs (4, 28–30). Patients carrying pathogenic HREs showed a higher rate of bulbar onset, earlier AAO, shorter survival, faster forced vital capacity (FVC) value decline, and a higher incidence of comorbid FTD and/or family history of dementia than those with normal genotypes (3, 32, 33). However, in our cohort, we found that patients with pathogenic HREs had a reduced median survival time, which was consistent with previous studies in China (6). Our result may be related to the low frequency of *C9orf72* HRE expansion mutations; large samples are needed to elucidate the potential associations between HREs in *C9orf72* and disease phenotypes. With regard to the clinical phenotypes of the six ALS patients with pathogenic HREs in *C9orf72*, only two presented memory impairment and abnormal behavior, while the remaining four showed pure ALS without memory impairment, which emphasizes the need to test for HREs in *C9orf72* in ALS patients with pure motor neuron signs.

**Figure 4:**
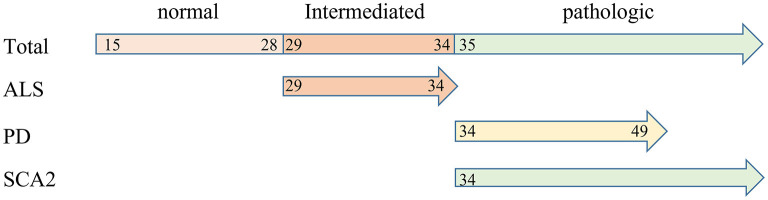
The spectrum of diseases associated with *ATXN2* polyQ repeat expansions.

To our knowledge, the normal CAG repeat size in *ATXN2* is 15–29 (34) and the different ranges of CAG repeat numbers contribute to different phenotypes (Figure 4), such as SCA2 (repeats >34), ALS (repeats: 29–34) and Parkinsonism (repeats: 34–49). To date, many previous studies have proposed that intermediate CAG repeats in *ATXN2* are associated with ALS (22, 31, 35–37). However, it is challenging to identify the risk range for *ATXN2* CAG repeat in ALS, and few studies reported a same risk range. Ming-Dong Wang et al. reported a intermediate size range of 30–33 (38). Meanwhile, Neuenschwander et al. (39) suggested a risk range of 29–32, 33 repeats. Subsequently, Sproviero et al. (31) reported the same results as reported by Neuenschwander et al. (39). In this study, we identified a cutoff value of 29 and found that intermediate repeats ranging from 29 to 34 were significantly associated with ALS. In addition, two studies have been suggested that shorter intermediate repeats may be a protective factor for ALS (31, 39), but there is still no consensus.

In SCA2, a strong inverse correlation between AAO and repeat length was observed (40). Other studies have indicated that patients with CAG repeat length >24 have an earlier AAO, but this was no significant in Chinese individuals (12, 34, 41). In our study, the patients with expanded CAG repeat length showed different manifestation. We found no association between clinical features and intermediate CAG repeat, which was in line with previous reports (31, 36, 41–45). We therefore propose that intermediate-length *ATXN2* repeats may be involved in ALS pathogenesis, but whether they influence the phenotypes of ALS remains to be determined.

In addition, we reviewed the clinical features of patients carrying intermediate CAG repeats (Supplementary Table 2). When comparing the patients with pathogenic CAG repeats (Supplementary Table 3), most patients with intermediate repeats in previous studies showed pure sporadic ALS or ALS with cognitive impairment, but few cases presented with ataxia (13, 31, 45–47). Moreover, in previous studies, patients with intermediate CAG repeats had an increased risk of combining with cognitive impairment or an FTD phenotype (43, 48). However, this phenomenon was not found in our 14 ALS patients with intermediate repeats. This discrepancy may arise from a difference in ethnic backgrounds. Additionally, some ALS patients were reported to carry full pathogenic CAG repeats (>34), and we also found two such ALS patients in our cohort. In summary, patients' clinical manifestations vary greatly (Supplementary Table 3): most patients carrying pathogenic repeats have a family history of SCA2 or ALS; a small portion of patients show pure ALS without ataxia; a few patients first present ataxia and gradually develop the ALS phenotype a few years after onset.

The genetic and clinical overlaps between ALS and SCA2 may arise from the common pathogenetic mechanisms of these two diseases. Many studies have found that CAG repeats in *ATXN2* specifically alter the secondary structure of the RNA, leading to toxic gain of function at the RNA level, which is also implicated in the pathogenesis of ALS (31). However, previous studies showed ALS patients have CAA interruption in the CAG repeat region (34), whereas SCA2 patients have pure CAG repeat regions (49). Pure CAG repeats form slippery hairpins, whereas the CAA interruptions determine the folding of the *ATXN2* transcript into branched hairpins which may hamper the interaction with double strand RNA-binding protein (34). This date may partly explain why *ATXN2* CAG repeats cause two different phenotypes. Meanwhile, TDP-43-positive inclusion bodies are the typical pathological manifestation of ALS. Many studies have found that ATXN2 can directly interact with TDP-43 and that expanded CAG repeats in *ATXN2* can promote cytoplasmic mis-localization of TDP-43 by enhancing C-terminal cleavage (9, 50). Further functional studies are needed to elucidate the pathogenic mechanisms of intermediate and pathogenic CAG repeats in the *ATXN2* gene in ALS.

Recently, a study showed that AR antagonists could accelerate disease onset in male *SOD1*^*G*93*A*^ ALS mice, leading to exacerbated muscle pathology (16), which indicates that AR may be implicated in the pathogenesis of ALS. However, we did not discover an association between CAG repeats in *AR* and ALS or its phenotypes in our cohort. In consisted with our results, many previous studies did not find an association between CAG repeats in *AR* and ALS either, even in other tissues, such as spinal cord and brain. Accordingly, it seems plausible to speculate that the lengths of CAG repeat in *AR* may not be a risk factor for ALS. More robust independent studies are still warranted to confirm this hypothesis.

Similar to *ATXN2, NOP56* is another causative gene of the SCA subtype, SCA36. Clinically, some SCA36 patients manifest the ALS phenotype, especially in cases reported in Japan. Genetically, some ALS patients carry pathogenic GGCCTG repeats in *NOP56* (18), suggesting an overlap of genetic and clinical manifestations between ALS and SCA36 (51, 52). Additionally, one study showed a progressive reduction in the NOP56 protein level in the large motor neurons of the *SOD1*^*G*93*A*^ ALS mouse model (19). However, our results found that the size of the GGCCTG repeat expansion in *NOP56* was not associated with ALS when a cutoff value of ≥9 was used and there was not a significant difference in the distribution of CAG repeats between patients and controls, which was consistent with the results of other studies (53, 54). In general, although ALS and SCA36 have similar clinical phenotypes, repeat expansions in *NOP56* might not be a risk factor for ALS. Thus, further understanding the normal functions of these genes will provide insight into their role in disease.

In conclusion, HREs in *C9orf72* are rare in ALS patients in mainland China, and GGCCTG repeats in *NOP56* and CAG repeats in *AR* may not be associated with an increased risk of ALS. Meanwhile, intermediate-length repeat expansions in *ATXN2* are more frequent in China, suggesting shared underlying etiological factors among neurodegenerative diseases. Our study has limitations. First, the number of the controls is less than half of ALS patients, which may affect the results of the statistical analysis. Second, we do not have a single control homogeneous group. Third, we did not evaluate the CAA interruptions in the CAG repeat region, which maybe significant to clarify the clinical manifestations of the two patients carrying 37 repeats.

## Data Availability Statement

The original contributions presented in the study are included in the article/Supplementary Material, further inquiries can be directed to the corresponding author.

## Ethics Statement

The studies involving human participants were reviewed and approved by the Ethics Committee and the Expert Committee of Xiangya Hospital, Central South University. The patients/participants provided their written informed consent to participate in this study.

## Author Contributions

XH and JW designed and conceptualized the study, analyzed the data, interpreted the data, drafted, and revised the manuscript. WL and PL play a major role in the acquisition of data and analyzed bioinformatic data. ZL, YY, and JN play a major role in the acquisition of data. LS and BT designed and conceptualized the study, analyzed the clinical data for diagnosis and differential diagnosis, and revised the manuscript. All authors contributed to the article and approved the submitted version.

## Funding

This work was supported by the National Key Research and Development Program of China (#2021YFA0805200); the National Major Projects in Brain Science and Brain-like Research (#2021ZD0201803); the National Key Research and Development Program of China (#2018YFC1312003); the Program of the National Natural Science Foundation of China (#82171431, 81671120, 81300981, and 81250015); the Natural Science Fund for Distinguished Young Scholars of Hunan Province, China (#2020JJ2057); the Project Program of National Clinical Research Center for Geriatric Disorders at Xiangya Hospital (#2020LNJJ13); the Degree and Postgraduate Education Reform Project of Central South University (#2020JGB136); and the Foundation of Xiangnan University (#N2020XJ88).

## Conflict of Interest

The authors declare that the research was conducted in the absence of any commercial or financial relationships that could be construed as a potential conflict of interest.

## Publisher's Note

All claims expressed in this article are solely those of the authors and do not necessarily represent those of their affiliated organizations, or those of the publisher, the editors and the reviewers. Any product that may be evaluated in this article, or claim that may be made by its manufacturer, is not guaranteed or endorsed by the publisher.
