# Supplementary material for: The Clinical and Polynucleotide Repeat Expansion Analysis of ATXN2, NOP56, AR and C9orf72 in Patients With ALS From Mainland China
Source: Front Neurol. 2022 May 6;13:811202. doi: 10.3389/fneur.2022.811202 (PMC9120572; doi:10.3389/fneur.2022.811202)
Supplement: Supplementary file 1 [file Data_Sheet_1.docx]

Supplementary Table 1. General features of ALS patients with intermediate-length repeats in *ATXN2* in our cohort.

| Patient | CAG repeat length | Sex | AAO (years) | Disease duration (months) | Site of onset | Family history | Characteristics | ALSFRS-R |
| --- | --- | --- | --- | --- | --- | --- | --- | --- |
| A0118 | 32 | M | 59 | 12^†^ | Spinal | Family history of ALS | ALS with ataxia | 38 |
| A0140 | 30 | M | 71 | 17^†^ | Spinal | Negative | Pure ALS | 38 |
| A22454 | 30 | M | 44 | 48^‡^ | Spinal | Negative | Pure ALS | 33 |
| A22522 | 31 | F | 52 | 7^‡^ | Spinal | Negative | Pure ALS | 27 |
| A27690 | 30 | M | 41 | 60^‡^ | Bulbar | Negative | Pure ALS | 43 |
| A28017 | 30 | M | 56 | 6 | Spinal | Negative | Pure ALS | 40 |
| A30686 | 31 | M | 59 | 5^‡^ | Bulbar | Negative | Pure ALS | 44 |
| A31776 | 30 | M | 46 | 151 | Spinal | Family history of ALS | Fasciculation for 10 years and gradual development of muscle weakness in all four extremities | 42 |
| A33439 | 31 | F | 47 | 60 | Spinal | Negative | Pure ALS | 43 |
| A36854 | 30 | M | 59 | 45 | Spinal | Negative | Pure ALS | 25 |
| A000631 | 30 | M | 68 | 44 | Spinal | Negative | Pure ALS | 27 |
| A000864 | 29 | F | 66 | 12 | Bulbar | Negative | Pure ALS | 36 |
| A001433 | 31 | F | 65 | 72 | Spinal | Negative | Pure ALS | 35 |
| A003580 | 31 | M | 41 | 12 | Spinal | Consanguineous family | Pure ALS | 41 |

^†^The patient was dead at the time of the study.

^‡^Lost to follow-up.

Supplementary Table 2. Clinical features of previously reported ALS patients with intermediate-lengths CAG repeats in *ATXN2.*

| Reference^†^ | CAG repeat length | AAO (years) | Disease duration (months) | Family history | Characteristics |
| --- | --- | --- | --- | --- | --- |
| ^1^ | 30 | 34 | 49.2 | NA | |
|  | 31 | 45 | 16.8 |  |  |
|  | 29 | 56 | 67.2 |  |  |
|  | 31 | 41 | 54 |  |  |
|  | 31 | 36 | 16.8 |  |  |
|  | 31 | 56 | 50.4 |  |  |
|  | 30 | 55 | 31.2 |  |  |
|  | 30 | 45 | 50.4 |  |  |
| ^2^ | 32 | 78 | 12 | NA | Pure ALS |
|  | 32 | 54 | NA |  | Pure ALS |
|  | 32 | 66 | NA |  | PMA |
|  | 32 | 79 | 24 |  | Pure ALS |
|  | 31 | 58 | 36 |  | Pure ALS |
|  | 31 | 58 | NA |  | Pure ALS |
|  | 31 | 38 | NA |  | Pure ALS |
|  | 32 | 52 | 14 |  | FTLD |
|  | 31 | 69 | NA |  | FTLD |
|  | 31 | 70 | NA |  | FTLD |
|  | 33 | 75 | NA | Family history of SCA | Pure ALS |
|  | 33 | 71 | NA |  | Pure ALS |
| ^3^ | 31 | 63 | 156 | Negative | Pure ALS (LMN-prevalent) |
|  | 31 | 66 | 22 | Negative | Pure ALS |
|  | 31 | 69 | 26 | Negative | Pure ALS |
|  | 32 | 74 | 30 | Negative | Pure ALS |
|  | 33 | 44 | 21 | Family history of PD | Pure ALS |
|  | 33 | 58 | 84 | Family history of PD | Pure ALS |
| ^4^ | 31 | 52 | 60 | NA | Pure ALS |
|  | 32 | 39 | 60 |  | Pure ALS |
|  | 32 | 77 | NA |  | Pure ALS |
|  | 32 | 8 | NA |  | Pure ALS |
| ^5^ | 32 | 52 | 9 | Negative | Pure ALS |
|  | 32 | 56 | 37 | Negative | Pure ALS |
|  | 32 | 42 | 35 | Negative | Pure ALS |
|  | 32 | 62 | 25 | Negative | Pure ALS |
|  | 32 | 69 | 38 | Negative | Pure ALS |
|  | 33 | 54 | 29 | Negative | Pure ALS |
|  | 33 | 35 | 13 | Negative | Pure ALS |
|  | 34 | 52 | 45 | Negative | Pure ALS |
|  | 33 | 75 | 59 | Family history of SCA | Pure ALS |
|  | 33 | 71 | 15 |  | Pure ALS |
| ^6^ | 29 | 31 | NA | Negative | Pure ALS |
|  | 29 | 41 |  | Negative | Pure ALS |
|  | 29 | 44 |  | Family history of ALS | Pure ALS |
|  | 30 | 46 |  | Negative | Pure ALS |
|  | 29 | 51 |  | Negative | Pure ALS |
|  | 30 | 54 |  | Negative | Pure ALS |
|  | 29 | 54 |  | Negative | Pure ALS |
|  | 29 | 56 |  | Negative | Pure ALS |
|  | 31 | 57 |  | Negative | Pure ALS |
|  | 33 | 60 |  | NA | Pure ALS |
| ^7^ | 30 | 75 | 96 | NA  Negative | Pure ALS |
|  | 29 | 67 | 12 |  | Pure ALS |
|  | 33 | 56 | 58 |  | ALS accompanied by mild cognitive impairment |
| ^8^ | 31 | 50 | 15 | Negative | Pure ALS |
|  | 30 | 57 | 76 | Negative | ALS accompanied by mild cognitive impairment |
|  | 33 | 45 | 55 | Negative | Pure ALS |
|  | 31 | 61 | 61 | Negative | Pure ALS |
|  | 31 | 40 | 15 | Negative | Pure ALS |
|  | 31 | 54 | 41 | Negative | Pure ALS |
|  | 29 | 67 | 12 | NA | Pure ALS |
| ^9^ | 30 | 75 | 96 | NA  Negative | Pure ALS |
|  | 33 | 60 | 60 |  | Pure ALS |
|  | 30 | 76 | NA |  | Pure ALS |
| ^10^ | 30 | 47 | NA | Negative | Pure ALS |
|  | 32 | 65 |  | Negative | Ataxia with ALS |
|  |  |  |  |  |  |

Abbreviation: NA: data not available; PMA: progressive muscular atrophy.

^†^ We did not summarize all the articles, as some of them did not describe the clinical data.

Supplementary Table 3. Clinical features of previously reported ALS patients with pathogenic CAG repeats in *ATXN2*.

| Reference | CAG repeat length | Diagnosis | AAO (years) | Disease duration (months) | Family history | Characteristics |
| --- | --- | --- | --- | --- | --- | --- |
| ^3^ | 37 | ALS | 60 | 48 | Negative | Pure ALS |
| ^2^ | 36 | ALS | 54 | 12 | NA | Pure ALS |
| ^5^ | 36 | ALS | 80 | 59 | Negative | Pure ALS |
|  | 39 | ALS | 71 | 15 | Negative |  |
|  | 35 | ALS | 52 | NA | Family history of SCA | The proband showed typical ALS.  Her sister, carrying a CAG repeat with a length of 34, showed ataxia without an ALS phenotype. |
| ^11^ | 39 | ALS | 62 | 23 | Family history of SCA | The proband showed typical ALS.  His niece, carrying a CAG repeat with a length of 40, showed ataxia without an ALS phenotype. |
| ^12^ | 37 | FTD-ALS | 76 | 60 | Family history of SCA | The proband showed typical FTD-ALS.  His niece carrying, a CAG repeat with a length of 37, showed ataxia without an ALS or FTD phenotype. |
| ^13^ | 39 | SCA-ALS | 55 | 168 | Family history of SCA | The proband showed ataxia and developed ALS 12 years after onset. |
| ^14^ | 35 | ALS | 41 | NA | | |
| ^15^ | 34–36 (4 patients) | ALS | NA | | | |
| ^16^ | 31–39 | FTD-ALS | NA | | | |
|  | 31–39 | ALS |  |  |  |  |
| ^17^ | 35 | ALS | NA | | | |

Abbreviation: NA: data not available.

References

1. Chen Y, Huang R, Yang Y, et al. Ataxin-2 intermediate-length polyglutamine: a possible risk factor for Chinese patients with amyotrophic lateral sclerosis. *Neurobiol Aging*. Oct 2011;32(10):1925 e1-5. doi:10.1016/j.neurobiolaging.2011.05.015

2. Ross OA, Rutherford NJ, Baker M, et al. Ataxin-2 repeat-length variation and neurodegeneration. *Hum Mol Genet*. Aug 15 2011;20(16):3207-12. doi:10.1093/hmg/ddr227

3. Corrado L, Mazzini L, Oggioni GD, et al. ATXN-2 CAG repeat expansions are interrupted in ALS patients. *Hum Genet*. Oct 2011;130(4):575-80. doi:10.1007/s00439-011-1000-2

4. Lahut S, Omur O, Uyan O, et al. ATXN2 and its neighbouring gene SH2B3 are associated with increased ALS risk in the Turkish population. *PLoS One*. 2012;7(8):e42956. doi:10.1371/journal.pone.0042956

5. Van Damme P, Veldink JH, van Blitterswijk M, et al. Expanded ATXN2 CAG repeat size in ALS identifies genetic overlap between ALS and SCA2. *Neurology*. Jun 14 2011;76(24):2066-72. doi:10.1212/WNL.0b013e31821f445b

6. Lu HP, Gan SR, Chen S, et al. Intermediate-length polyglutamine in ATXN2 is a possible risk factor among Eastern Chinese patients with amyotrophic lateral sclerosis. *Neurobiol Aging*. Mar 2015;36(3):1603 e11-4. doi:10.1016/j.neurobiolaging.2014.10.015

7. Tan RH, Kril JJ, McGinley C, et al. Cerebellar neuronal loss in amyotrophic lateral sclerosis cases with ATXN2 intermediate repeat expansions. *Ann Neurol*. Feb 2016;79(2):295-305. doi:10.1002/ana.24565

8. Kim YE, Oh KW, Noh MY, et al. Analysis of ATXN2 trinucleotide repeats in Korean patients with amyotrophic lateral sclerosis. *Neurobiol Aging*. Jul 2018;67:201 e5-201 e8. doi:10.1016/j.neurobiolaging.2018.03.019

9. Yang Y, Halliday GM, Kiernan MC, Tan RH. TDP-43 levels in the brain tissue of ALS cases with and without C9ORF72 or ATXN2 gene expansions. *Neurology*. Nov 5 2019;93(19):e1748-e1755. doi:10.1212/WNL.0000000000008439

10. Ghahremani Nezhad H, Franklin JP, Alix JJP, et al. Simultaneous ALS and SCA2 associated with an intermediate-length ATXN2 CAG-repeat expansion. *Amyotroph Lateral Scler Frontotemporal Degener*. Dec 7 2020:1-3. doi:10.1080/21678421.2020.1853172

11. Tazen S, Figueroa K, Kwan JY, et al. Amyotrophic lateral sclerosis and spinocerebellar ataxia type 2 in a family with full CAG repeat expansions of ATXN2. *JAMA Neurol*. Oct 2013;70(10):1302-4. doi:10.1001/jamaneurol.2013.443

12. Baumer D, East SZ, Tseu B, et al. FTLD-ALS of TDP-43 type and SCA2 in a family with a full ataxin-2 polyglutamine expansion. *Acta Neuropathol*. Oct 2014;128(4):597-604. doi:10.1007/s00401-014-1277-z

13. Chiò A, Calvo A, Moglia C, et al. ATXN2 polyQ intermediate repeats are a modifier of ALS survival. *Neurology*. Jan 20 2015;84(3):251-8. doi:10.1212/wnl.0000000000001159

14. Gispert S, Kurz A, Waibel S, et al. The modulation of Amyotrophic Lateral Sclerosis risk by ataxin-2 intermediate polyglutamine expansions is a specific effect. *Neurobiol Dis*. Jan 2012;45(1):356-61. doi:10.1016/j.nbd.2011.08.021

15. Conforti FL, Spataro R, Sproviero W, et al. Ataxin-1 and ataxin-2 intermediate-length PolyQ expansions in amyotrophic lateral sclerosis. *Neurology*. Dec 11 2012;79(24):2315-20. doi:10.1212/WNL.0b013e318278b618

16. Lattante S, Millecamps S, Stevanin G, et al. Contribution of ATXN2 intermediary polyQ expansions in a spectrum of neurodegenerative disorders. *Neurology*. Sep 9 2014;83(11):990-5. doi:10.1212/WNL.0000000000000778

17. Tavares de Andrade HM, Cintra VP, de Albuquerque M, et al. Intermediate-length CAG repeat in ATXN2 is associated with increased risk for amyotrophic lateral sclerosis in Brazilian patients. *Neurobiol Aging*. Sep 2018;69:292 e15-292 e18. doi:10.1016/j.neurobiolaging.2018.04.020
